# Development and multicenter validation of an explainable machine learning diagnostic criteria for pediatric abdominal sepsis

**DOI:** 10.1038/s41746-026-02500-0

**Published:** 2026-03-03

**Authors:** Suqi Cao, Duote Cai, Shuhao Zhang, Yuchen He, Xiaojian Yuan, Zhiqiang Zhu, Xuefeng Miao, Shannan Wu, Yongxing Zhong, Fangyan Yang, Guofeng Yin, Juying Yan, Junjie Chen, Donglai Hu, Menglu Yu, Zhijian Zhou, Qiongjie Ruan, Boyun Xuan, Yihao Cai, Liangting Tao, Weiwei Zhang, Ting Yu, Junfen Zhou, Wei Song, Yanwei Tong, Yanhui Tian, Chunting Zhou, Dingfeng Wu, Daqing Ma, Zhigang Gao

**Affiliations:** 1 National Clinical Research Center for Child And Adolescents’ Heath and Diseases, The Children’s Hospital, Zhejiang University School of Medicine, Hangzhou, China; 2 General Surgery Department, Children’s Hospital, Zhejiang University School of Medicine, Hangzhou, PR China; 3 Yiwu Maternity and Children Hospital, Yiwu, China; 4 Shaoxing Maternal and Child Health Care Hospital, Shaoxing, PR China; 5 Department of Pediatric Surgery, Jinhua Maternal and Child Health Care Hospital, Jinhua, China; 6 Zhuji Maternity and Child Health Hospital, Zhuji, China; 7 Wenling Maternal and Child Health Care Hospital, Wenling, PR China; 8 Quzhou Maternal and Child Health Care Hospital, Quzhou, China; 9 Perioperative and Systems Medicine Laboratory and Department of Anesthesiology, National Clinical Research Center for Child and Adolescents’ Heath and Diseases, Children’s Hospital, Zhejiang University School of Medicine, Hangzhou, China; 10 Division of Anaesthetics, Pain Medicine and Intensive Care, Department of Surgery and Cancer, Faculty of Medicine, Imperial College London, Chelsea and Westminster Hospital, London, UK

**Keywords:** Computational biology and bioinformatics, Diseases, Health care, Medical research

## Abstract

Accurate identification of early pediatric abdominal sepsis (PAS) is essential to improving outcomes, yet most existing pediatric sepsis criteria and scoring tools primarily focus on cardiopulmonary dysfunction and overlook early intra-abdominal infections. To address this gap, we combined the real-world data with explainable machine learning to develop the Abdominal Sepsis Diagnosis model (ABSeD) for clinical decision support. The model construction used the retrospective data from 6566 pediatric patients who were admitted to the Children’s Hospital, Zhejiang University School of Medicine from 2019 to 2023. Prospective data from 308 recruited patients across seven independent hospitals collected between January and March 2025 served as an external validation cohort. PAS status was determined through consensus or by reviewing laparoscopic surgery records. Multiple machine learning algorithms were compared, and the optimal model was further refined by hyper-parameter tuning. The ABSeD model, integrating nine routine clinical variables, demonstrated high diagnostic accuracy (training set: AUC = 0.934, 95% CI: [0.912, 0.950]; accuracy = 0.870, precision = 0.910), and robust multicenter generalizability (AUC = 0.928, 95% CI: [0.895, 0.961]; accuracy = 0.873, precision = 0.924). This model offers an explainable and practical digital tool for early detection of PAS, with potential to enhance timely intervention in hospitalized children with suspected or clinically identified intra-abdominal septic pathology.

## Introduction

Sepsis is one of the leading causes of death in intensive care units^1,2^. Globally, approximately 25 million children are affected by sepsis annually and account for half of the global incidence of sepsis cases^3^. The mortality is even higher in children under 14 years of age^3^. The medical and economic burden of childhood sepsis poses a considerable challenge to societies and families worldwide, particularly in developing countries^4^.

Three iterations of the sepsis diagnostic and treatment guidelines (i.e., Sepsis 1.0^5^, 2.0^6^, and 3.0^2^) are available thus far. However, Sepsis 1.0 has been criticised for its lack of specificity and the potential for over-medicalization^7^. Although the subsequent update, Sepsis 2.0, enhanced the accuracy of clinical diagnosis, its complexity hinders practical application in clinical settings^6^. The most recent iteration, Sepsis 3.0 introduced significant amendments but these are mostly centered on organ failure^2^. Once the patient reaches to the organ failure stage, treatment is often ineffective. Moreover, most diagnostic tools developed based on adult criteria have limited applicability in children due to the significant disparities in pathophysiology^8^. Therefore, there is an urgent need to develop a diagnostic tool for pediatric use.

Intra-abdominal infection is one of leading causes of sepsis in children and leads to damage vital organs such as the liver, pancreas and gastrointestinal tract; there is very poor prognosis^9^. The existing pediatric organ function damage severity score assays such as the pediatric Sequential Organ Failure Assessment (pSOFA) scores^10^ primarily focuses on damage to cardiopulmonary function. Unfortunately, in clinical practice, when sepsis-like abnormalities emerge in cardiopulmonary indicators, the damage to other vital organs has already reached a very severe or even irreversible state. While the phoenix criteria developed by Sanchez-Pinto et al. are expected to enhance the accuracy of diagnosing sepsis in children^11^, it is not sufficiently sensitive for early diagnosis of pediatric abdominal sepsis (PAS) disease in clinical practice^8^. Hence, there is an urgent need for objective, high-quality and unified PAS diagnostic criteria in clinical practice.

Machine learning is becoming useful for early identification and management of diseases. A substantial body of evidence showed the superiority of a machine learning derived scoring system over traditional methods^12,13^. However, due to inherent challenges including a paucity of pediatric medical data^14^, machine learning derived models developed based on traditional clinical biomarkers lack specificity^15^. Conversely, while multi-omics integration strategies have potential to enhance diagnostic efficiency^16^, genetic profiling offers limited clinical interpretability and remains incompatible with hospital information systems^17^.

Thus, this study aims to retrospectively analyze data from 6,566 pediatric patients with various abdominal disease conditions who were hospitalized in the Children’s Hospital, Zhejiang University School of Medicine between 2019 and 2023. The primary aim is to develop a clinically applicable diagnostic tool for PAS. This will assist clinicians in timely identification of children who are at a high risk of PAS and subsequently improve patient care. The generalizability of the model was verified with data collected prospectively from seven hospitals in Zhejiang Province, China from January to March of 2025.

## Results

### Participants and data source

**Fig. 1:**
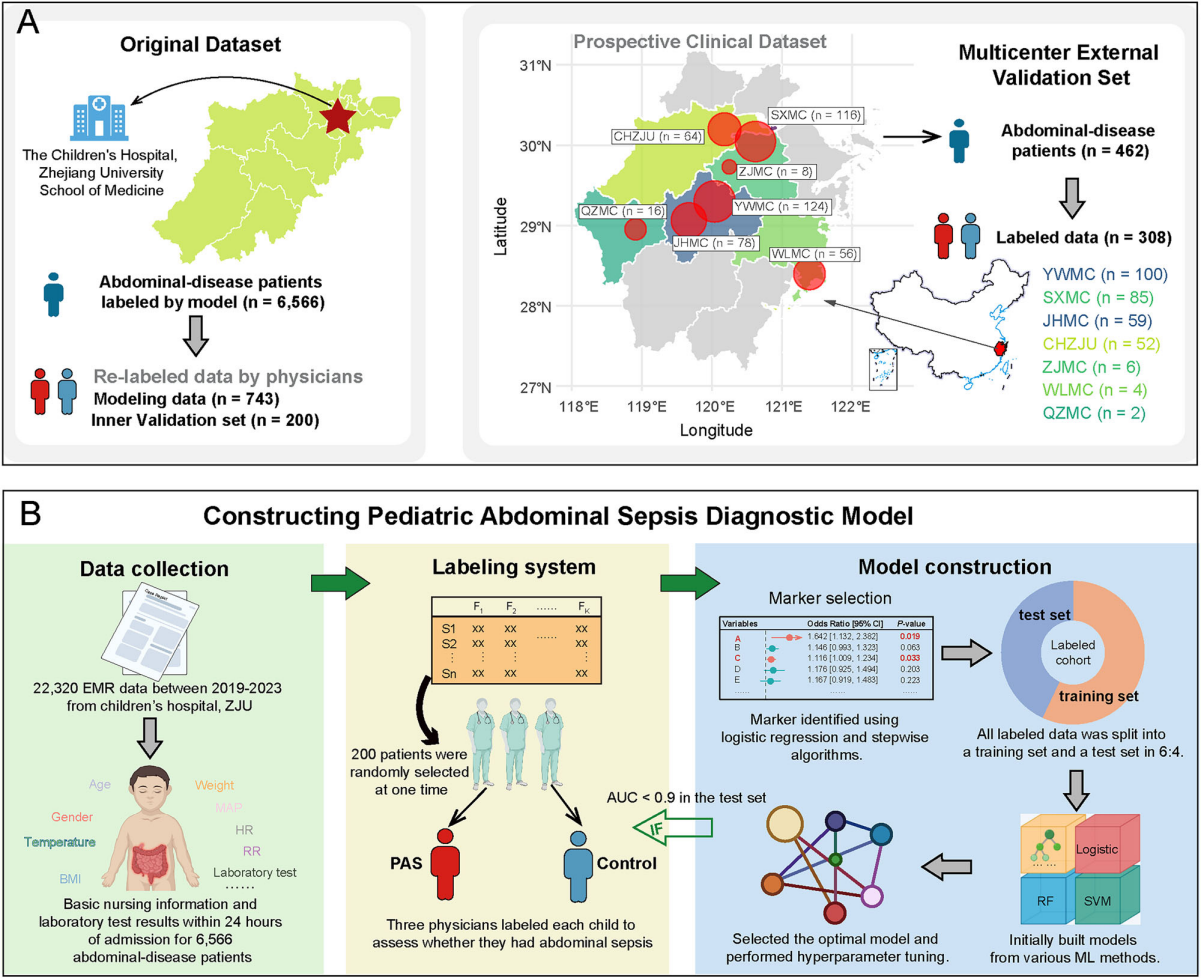
Data distribution and flowchart for constructing the pediatric abdominal sepsis (PAS) diagnostic model, namely ABSeD. **A** Distribution of the original data set (left) used for modeling and the multicenter external validation set (right) collected by the diagnostic platform. **B** Data set was derived from the electronic medical records of patients admitted to Children’s Hospital, Zhejiang University School of Medicine from 2019 to 2023. Three physicians utilized the annotation system to diagnose sepsis in 200 children with abdominal diseases in each round. The labeled data were then divided into training and test sets at a ratio of 6:4. The PAS diagnostic model was developed within the training set, employing various machine learning (ML) methodologies. The algorithm demonstrating optimal performance was then selected for hyper-parameter tuning. When the diagnostic model established by the optimal algorithm has an AUC ≥ 0.9 in the test set, labeling is terminated. Otherwise, the subsequent round of labeling is performed. Some elements of this figure were obtained from Biorender (KR29AUPZG4). Created in BioRender. Team, Z. (2026) https://BioRender.com/qx7rnwq.

Data were obtained from the electronic medical records of 22,320 children who visited the Children’s Hospital, Zhejiang University School of Medicine from 2019 to 2023. Those data were routine blood, blood gas and electrolytes, biochemical and coagulation spectrum. Subsequently, after excluding children under 1 year old, a total of 6566 children with abdominal diseases including intestinal obstruction, megacolon, oblique hernia or appendicitis were screened by attending and/or senior consultant physicians (Fig. 1A, B). To ensure validity, all data utilized for screening and model construction underwent labeling (see below) by three attending or senior physicians.

In accordance with our diagnostic model, a user-friendly platform (http://www.zjuchsep.com/absed) was developed and used to recruit prospective cases from seven hospitals in Zhejiang Province, China from January to March 2025. During this period, 462 cases were entered into the platform. Of those, 308 children (80 PAS and 228 controls) underwent surgical or follow-up confirmation of their PAS status, and these cases were used as a multicenter external validation set to evaluate the diagnostic ability of the model (Fig. 1A).

### Nine biomarkers identified for the diagnosis of Pediatric Abdominal Sepsis (PAS)

**Fig. 2:**
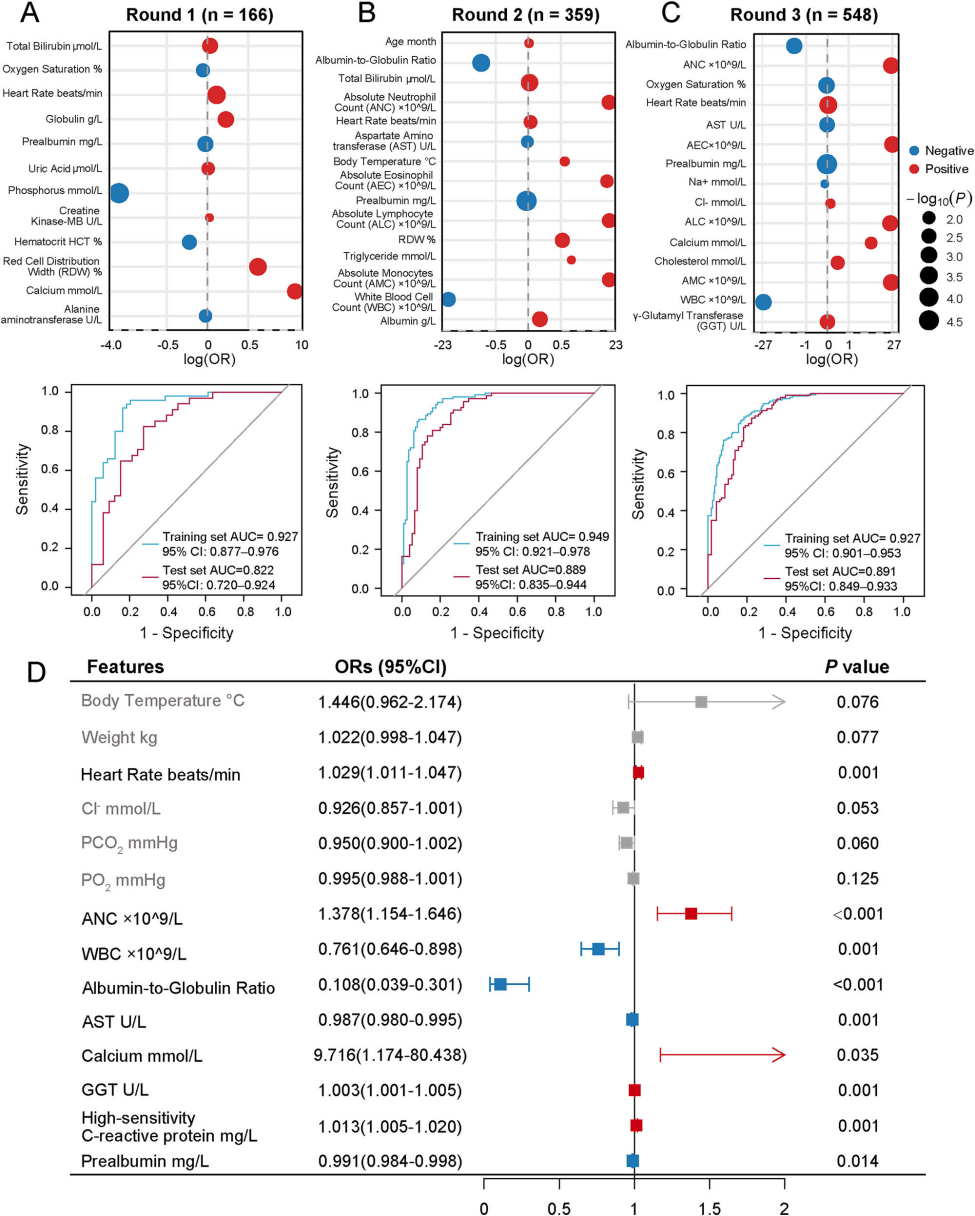
Overview of marker screening. **A** Dot plot of significant indicators and the receiver operating characteristic (ROC) curves for the optimal model in round 1. The position along the y-axis corresponds to the log-transformed odds ratio (log(OR)), indicating the direction and magnitude of association with the outcome, and dot size is proportional to the –log₁₀(*P* value), highlighting more statistically significant variables; The blue curve signified the AUC performance of the optimal model in the training set, while the purple curve denoted the AUC performance in the test set. The same analysis of round 2 and round 3 is shown in (**B**–**C**). **D** Forest plot presents the odds ratios (ORs) and 95% confidence intervals (CIs) of markers calculated by multivariate logistic regression and stepwise analysis.

Following the initial round, which involved the removal of cases exhibiting inconsistent doctors’ labeling and excessive missing values, a total of 166 cases were retained for the subsequent selection of indicators and model construction. A total of 12 differential indicators were identified and the optimal model exhibited overfitting tendencies in this round, as shown by an AUC = 0.927 (95% CI: [0.877, 0.976]) in the training set and an AUC = 0.822 (95% CI: [0.720, 0.924]) in the test set (Fig. 2A). Subsequent incorporation of the next two rounds of labeling results led to an enhancement in the performance of the model; nonetheless, this did not attain the predetermined threshold. The AUC of the optimal model in the second round was 0.949 (95% CI: [0.921, 0.978]) in the training set and 0.889 (95% CI: [0.835, 0.944]) in the test set, while the AUC in the third round was 0.927 (95% CI: [0.901, 0.953]) in the training set and 0.891 (95% CI: [0.849, 0.933]) in the test set (Fig. 2B, C).

In the subsequent fourth round of labeling, 800 pediatric patients with abdominal diseases were relabeled whether they had sepsis. The study population included 743 children who met the established inclusion criteria, with a median age of 62 months [IQR: 19–108.5] and 60.027% (446 cases) were male. Of these 313 cases were PAS and 430 cases were classified as other abdominal diseases (control group). Supplementary Table 1 presents a comprehensive overview of the basic nursing and laboratory test data. Supplementary Table 2 further characterized adjudicated PAS cases versus controls using objective clinical anchors (surgery, prolonged escalation antibiotics >7 days, intra-abdominal abscess, and hospital stay time), which were reported for transparency but not used as model inputs. To identify potential risk factors associated with PAS, a univariate logistic regression analysis was performed. This analysis revealed 39 parameters that may serve as potential risk markers (*P* < 0.05; Supplementary Fig. 1 and Supplementary Table 3).

Final PAS diagnostic factors were determined by performing multivariate logistic regression and stepwise analysis (Fig. 2D). Heart rate (OR = 1.029, 95% CI: [1.011, 1.047], *P* = 0.001), absolute neutrophil count (ANC; OR = 1.378, 95% CI: [1.154, 1.646], *P* < 0.001), white blood cell count (WBC; OR = 0.761, 95% CI: [0.646, 0.898], *P* = 0.001), albumin-to-globulin ratio (A/G; OR = 0.108, 95% CI: [0.039, 0.301], *P* < 0.001), aspartate aminotransferase (AST; OR = 0.987, 95% CI: [0.980, 0.995], *P* = 0.001), calcium (OR = 9.716, 95% CI: [1.174, 80.438], *P* = 0.035), γ-glutamyl transferase (GGT; OR = 1.003, 95% CI: [1.001, 1.005], *P* = 0.001), high-sensitivity C-reactive protein (hs-CRP; OR = 1.013, 95% CI: [1.005, 1.020], *P* = 0.001) and prealbumin (PA; OR = 0.991, 95% CI: [0.984, 0.998], *P* = 0.014) were significantly associated with PAS.

### Random forest algorithm demonstrated superior performance and stability in PAS diagnostic capability

**Fig. 3:**
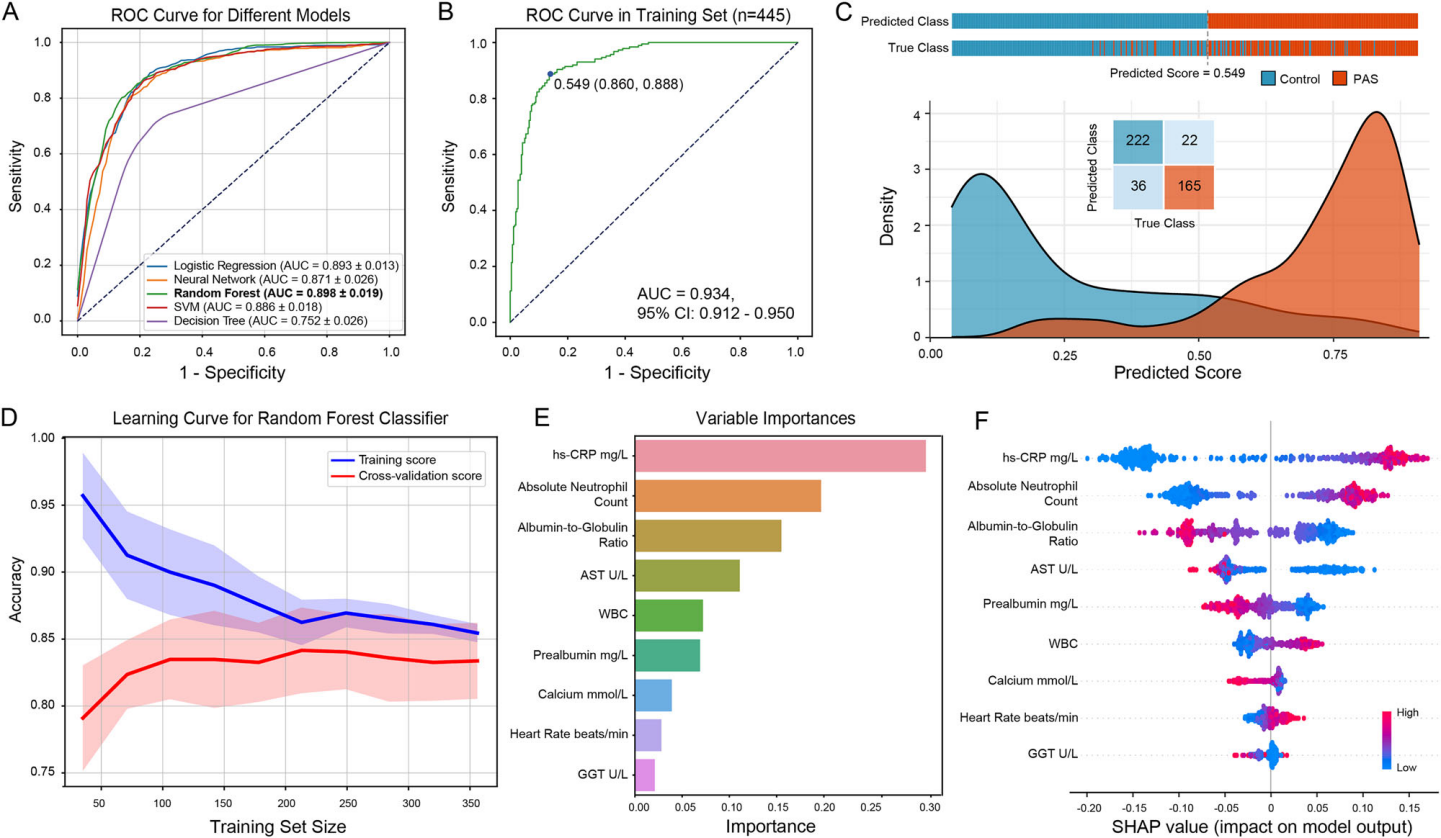
Model construction and performance evaluation. **A** Receiver operating characteristic (ROC) curves for five common machine learning models. **B** Area under the ROC curve (AUC) was used to diagnose PAS. The optimal cut-off value of the model predicted score was 0.549, corresponding to a sensitivity of 0.888 and a specificity of 0.860. **C** Distribution of predicted scores and confusion matrix of ABSeD model in the PAS and control groups in the training set. **D** Learning curve showed the performance of the ABSeD random forest classifier with different training set sizes. **E** Relative importance of each feature in the construction of the ABSeD model. **F** SHapley Additive exPlanations (SHAP) values provide an indication of the contribution of each feature to the prediction results. hs-CRP high-sensitivity C-reactive protein, AST aspartate aminotransferase, WBC white blood cell count, GGT γ-glutamyl transferase.

Subsequently, all 743 labeled data sets were divided into training (187 PAS and 258 controls) and test sets (126 PAS and 172 controls) in a ratio of 6:4 using a random stratified sampling method. Based on the nine biomarkers described above, the default parameters of five widely utilized machine learning methods were employed to construct a PAS diagnostic model in the training set. As demonstrated in Fig. 3A, the random forest model (AUC = 0.898 ± 0.019) exhibited superior performance in PAS recognition when compared to alternative models (Logistic Regression: AUC = 0.893 ± 0.013; Neural Network: AUC = 0.871 ± 0.026; SVM: AUC = 0.886 ± 0.018; Decision Tree: AUC = 0.752 ± 0.026). After hyper-parameter tuning, the final abdominal sepsis diagnosis (ABSeD) model using the random forest algorithm was identified for predicting the occurrence of abdominal sepsis. ROC analysis demonstrated that the model exhibited high predictive capability in diagnosing PAS, with an AUC of 0.934 (95% CI: [0.912, 0.950]; Fig. 3B). Concurrently, the model allocated a predicted score to each patient, denoting the probability that the model predicts a given patient as PAS. Furthermore, the optimal cut-off value was 0.549.

Distribution of predicted scores for the PAS model was shown in Fig. 3C with a model precision of 0.910 and an accuracy of 0.870; 88.235% of PAS children were correctly identified. The learning curve suggested that the ABSeD model exhibits adequate stability (Fig. 3D). The predicted score of the PAS group was significantly increased compared to control (*P* < 0.001; Supplementary Fig. 2A). In addition, with the exception of heart rate (*P* = 0.830), there were statistically significant differences between the PAS and control groups with regard to the other markers (*P* < 0.001; Supplementary Fig. 2A). Among them, hs-CRP was the most significant indicator for constructing the ABSeD model and an important contributing factor for predicting diagnostic outcomes (Fig. 3E, F).

### ABSeD model demonstrated robust generalization

**Fig. 4:**
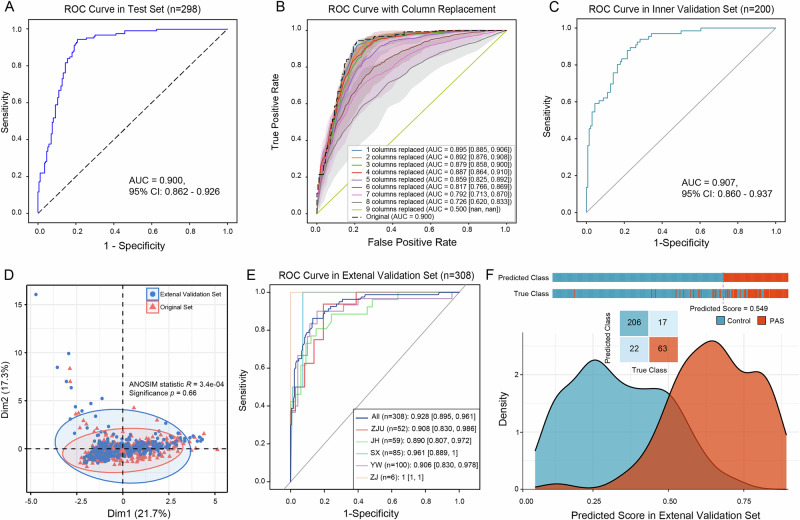
Verification of ABSeD model generalization ability. **A** Receiver operating characteristic (ROC) curve in the test set. **B** ROC curves with column values randomly replaced by the mean of the corresponding training set column. **C** ROC curve in the inner validation set. **D** Comparison of intergroup differences between external validation set and the original data set. **E** ROC curves in the multicenter external validation set. **F** Distribution of predicted scores and confusion matrix of ABSeD model in the PAS and control groups in the external validation set. ZJU: Children’s Hospital Zhejiang University School of Medicine; JH: Jinhua Maternal and Child Health Care Hospital; SX: Shaoxing Maternal and Child Health Care Hospital; YW: Yiwu Maternal and Child Health Care Hospital; ZJ: Zhuji Maternal and Child Health Care Hospital.

To assess the capacity for stability and generalization of the ABSeD model, its performance was evaluated on the test, internal and multicenter external validation sets, respectively. ABSeD model exhibited adequate PAS diagnostic capability in the test set, with an AUC value of 0.900 (95% CI: [0.862, 0.926]) (Fig. 4A). Similar to the results of the training set, with the exception of heart rate (*P* = 0.370) other factors exhibited significant differences between the PAS and the control groups (*P* < 0.001; Supplementary Fig. 2B). Following implementation of the 0.549 threshold, results of the confusion matrix indicated that 90.476% of the PAS children were accurately identified with the model’s precision of 0.921 and an accuracy of 0.849 (Supplementary Fig. 3A). In particular, to explore the impact of feature loss on model performance, the columns in the test set were randomly replaced with the average values of the corresponding indicators in the training set. The findings indicated that in the extreme noise scenario (i.e., the six markers in the test set are substituted with the mean values), the AUC of the model can still reach 0.817 (95% CI: [0.766, 0.869]) (Fig. 4B).

The internal validation set was derived from data of 200 children (66 PAS and 134 controls) diagnosed with abdominal diseases who were randomly selected from the original unlabeled dataset. These data were labeled by three attending or higher-level physicians. The ABSeD model demonstrated superior PAS diagnostic performance in the internal validation set (AUC = 0.907, 95% CI: [0.860, 0.937]) (Fig. 4C). Confusion matrix results further demonstrated that 86.364% of PAS patients were accurately identified and the model exhibited a precision of 0.921 and an accuracy of 0.810 (Supplementary Fig. 3B).

The performance of the ABSeD model was further evaluated using a multicenter external validation set. The absence of a batch effect between the external validation set and the original dataset was confirmed (*P* = 0.660; Fig. 4D). The model exhibited remarkable PAS diagnostic capability on the external validation set (AUC = 0.928, 95%CI: [0.895, 0.961]; Fig. 4E). With the exception of two hospitals that only had PAS or control cases, the model demonstrated exceptional diagnostic performance in the remaining five hospitals (Fig. 4E). In addition, a substantial disparity was observed in each index between the PAS and the control groups (Calcium: *P* = 0.011; GGT: *P* = 0.048; others: *P* < 0.001; Supplementary Fig. 4). The Fig. 4F illustrates distribution of predicted scores in multicenter external validation set. Outcomes of the confusion matrix indicated that the overall precision of the model was 0.924 and its accuracy was 0.873. The detailed results from different hospitals was presented in Supplementary Table 4.

### Application of the ABSeD model to facilitate clinical diagnosis

**Fig. 5:**
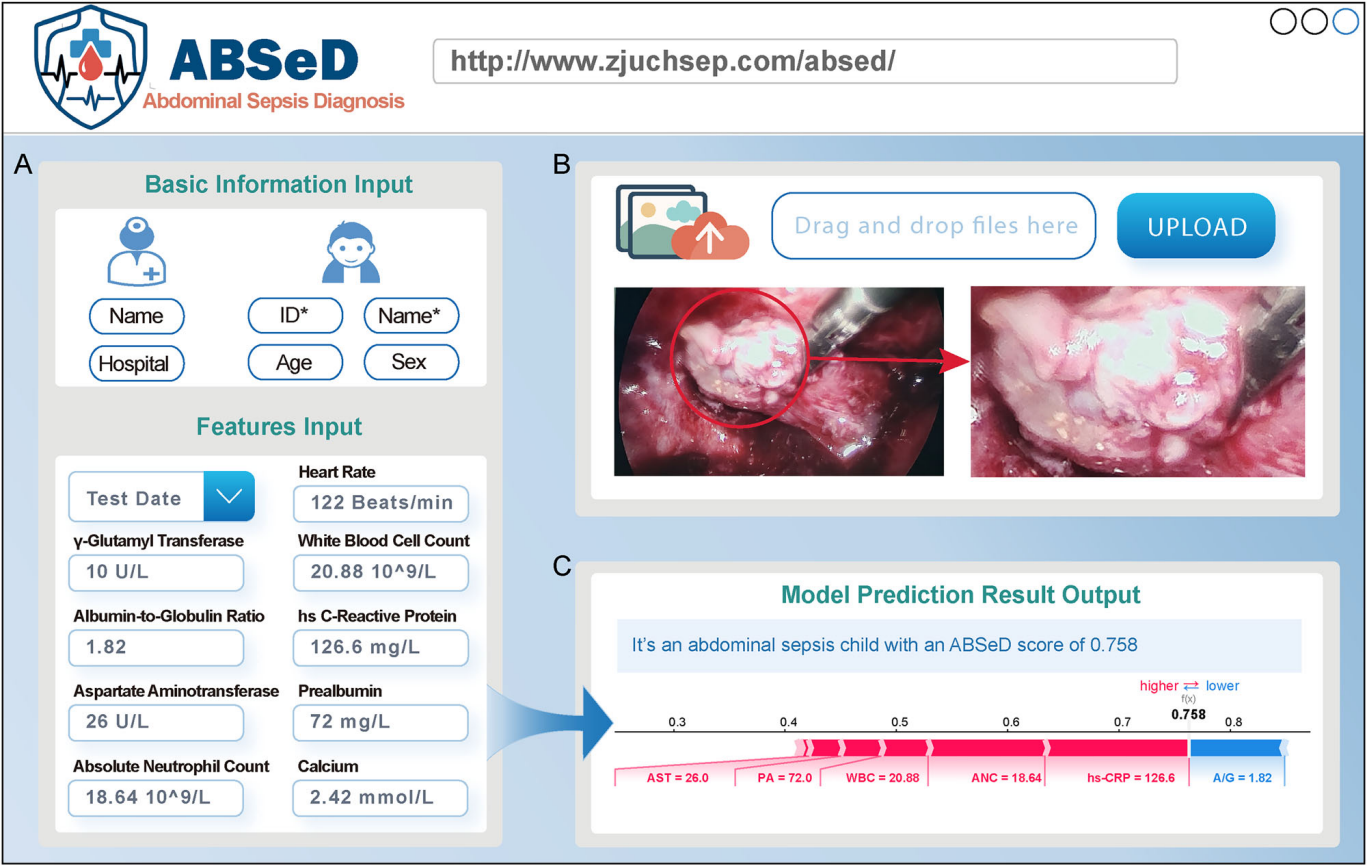
Introduction to the diagnostic platform for PAS. **A** Information section including basic information input and features input. **B** Image upload is an optional section; can be done separately by users. **C** Result output includes prediction scores, diagnostic results and model interpretability results. AST aspartate aminotransferase, PA prealbumin, WBC white blood cell count, ANC absolute neutrophil count, hs-CRP high-sensitivity C-reactive protein, A/G albumin-to-globulin ratio.

To facilitate clinical use, a user-friendly sepsis diagnostic platform was developed (http://www.zjuchsep.com/absed). This platform comprises three sections: information input (Fig. 5A), image input (Fig. 5B) and result output (Fig. 5C). First, the user must enter basic information, including the data source (e.g., hospital or region), the patient’s gender and age. Subsequently, the user is required to input the patient’s test time and the test results of nine sepsis diagnostic markers (Fig. 5A). The platform requires users to enter numeric laboratory values. If the hs-CRP test result is below the detection limit (e.g., "<0.5 mg/L"), a value of 0.1 is entered into the platform for model calculation. Non-numeric entries are recorded as missing. During inference, missing values are filled using the corresponding mean values derived from the internal training dataset before generating ABSeD predictions. Image upload is an optional section and can be done separately after surgery. However, it is important to note that if a patient has previously undergone a diagnostic evaluation on the website before undergoing surgery and only the image information has been uploaded postoperatively, it is essential to ensure that the patient ID remains consistent with the ID that was previously entered (Fig. 5B).

For example a patient had preoperative heart rate, GGT, WBC, A/G, hs-CRP, AST, PA, ANC and calcium of 122 beats/min, 10 U/L, 20.88 10^9/L, 1.82, 126.6 mg/L, 26 U/L, 72 mg/L, 18.64 10^9^/L and 2.42 mmol/L, respectively. Following a diagnosis by the platform, the given patient’s predicted score was 0.758, which exceeded the established threshold of 0.549. Consequently, the ABSeD model diagnosed the given patient with PAS. Our platform provided both a predicted score and interpretable results derived from the ABSeD model. As shown in Fig. 5C, hs-CRP emerged as the most important contributor for the model to diagnose that a given child has PAS; this was followed by ANC and WBC. Subsequent surgery confirmed that this was indeed a child with PAS (Fig. 5B).

## Discussion

Sepsis-induced refractory shock and multiple organ dysfunction persist as primary causes of infection-related mortality in children, imposing a significant global health burden^4^. Early diagnosis is critical for improving outcomes, yet current pediatric sepsis guidelines lack universal applicability and clinical decision-making remains largely dependent on clinicians’ judgment^18^. In this study, we developed an interpretable, high-performance early diagnostic model for PAS by integrating EMR data with multiple machine learning methods, and translated it into the clinically applicable ABSeD platform (http://www.zjuchsep.com/absed).

The present study identified nine key clinical biomarkers for PAS diagnosis, including heart rate, GGT, WBC, A/G ratio, hs-CRP, AST, prealbumin (PA), ANC and calcium. Among them, heart rate showed the greatest sensitivity in predicting the severity of infection^19^. And it has been employed as a biomarker for early prediction of neonatal sepsis in previous studies^20^. WBC, ANC and CRP are indicators of the inflammatory response and immune status and form the basis for clinical sepsis screening^21^. Furthermore, hs-CRP was reported to exhibit superior diagnostic accuracy in pediatric sepsis when compared with other markers^22^. The remaining factors primarily reflect sepsis-induced organ dysfunction. For example, there is a statistically significant association between GGT and pediatric sepsis^23^. Moreover, hypocalcemia (i.e., low serum ionized calcium level) was found to be prevalent in neonatal sepsis and is significantly associated with mortality^24^. PA was also reported to be the most sensitive predictor of sepsis-related mortality^25^.

Although these markers are not exclusive to sepsis and there is patient variability^26^, machine learning is a quantitative approach that can be used to implement sepsis prediction algorithms by integrating biomarkers^17^. A meta-analysis of 76 machine learning models for the diagnosis of pediatric sepsis revealed that the random forest algorithm was the most widely adopted^27^. Consistent with this, after comparing multiple commonly used machine learning methods, our study found that the random forest was the optimal approach for constructing a PAS diagnostic tool (ABSeD). The majority of diagnostic models for pediatric sepsis were developed without multicenter external validation so generalizability is limited^13,27^. The ABSeD platform is capable of not only generating a prediction of PAS at an early stage but also producing a model score and SHAP interpretable outputs. These outputs are intended to assist physicians in evaluating the severity of PAS and identifying clinical indicators that require prioritization. By March 2025, the platform had prospectively accrued 462 entries from seven hospitals. Cases with definitive outcome ascertainment through surgery and/or follow-up (n = 308) were included as the multicenter external validation cohort, in which the model demonstrated robust prediction (AUC = 0.928, accuracy = 0.873, precision = 0.924).

The present study has several notable strengths. The selection of interpretable clinical parameters resulted in enhanced transparency and clinical relevance of the diagnostic model. Prospective multicenter external validation supports the feasibility and diagnostic performance of ABSeD in the intended-use population. Ultimately, development of the ABSeD platform facilitates real-world clinical integration. Nevertheless, several limitations should be acknowledged. Firstly, inflammatory mediators have been proposed as biomarkers for sepsis diagnosis^17^, but they were not incorporated in the present study due to missing data. Secondly, the model is designed for early detection of PAS and may be less suitable for identifying late-stage sepsis, which is characterized by immunosuppression (e.g., leukopenia or neutropenia)^28,29^. Thirdly, the usefulness of the cross-ethnic and cross-regional applicability of the model is unknown. Fourthly, ABSeD is not intended as a general sepsis screening tool for unselected pediatric populations, because it was developed for children with suspected or clinically identified intra-abdominal pathology. Finally, discordant physician-labeled cases were excluded during iterative labeling as a noise-control strategy, which may under-represent diagnostically ambiguous borderline cases. All these issues warrant further investigation.

In summary, we developed and externally validated the ABSeD model. This is an interpretable, high-performance machine learning tool for the early diagnosis of PAS. The model incorporated nine common clinical biomarkers: heart rate, GGT, WBC, A/G ratio, hs-CRP, AST, prealbumin, ANC and calcium. These variables are routinely measured and therefore support practical deployment within abdominal-disease care pathways. Construction of the ABSeD platform facilitates real-world clinical implementation that aims to assist clinicians in improving the early recognition and timely intervention for pediatric abdominal sepsis.

## Methods

### Data processing and feature extraction

Preliminary features were derived from demographic and laboratory data including blood routine, blood gas and biochemistry within 24 hours of admission. For variables measured multiple times within 24 hours of admission, we used the first available value within the window as the model input to reflect early risk assessment. Features with missing values greater than 20% were excluded and the remaining missing values were substituted with the mean value of the corresponding features. Univariate logistic regression was carried out to identify factors that exhibited significant differences between the PAS group and control group. To further enhance computational efficiency of the model and explanatory power, multivariate logistic regression and stepwise regression algorithms were employed to identify the final difference features. These features served as diagnostic biomarkers for PAS.

### Labeling system based on active learning

Machine learning requires a large amount of labeled data for the model construction. To address the time-consuming nature of labeling, we have developed a labeling system based on active learning. Specifically, we started by using a small number of manually labeled results to build a preliminary model and then used that model to predict the case status (PAS or control) in the unlabeled dataset. Subsequently, an equivalent number of children in the PAS and control groups predicted by the model were randomly selected from the unlabeled dataset through a stratified sampling algorithm. Next, they were returned for manual confirmation and review. Accordingly, the manually labeled results are returned to the model for the next iteration which updated the algorithm and hyper-parameters to improve predictive performance. This gradual improvement process is repeated until the performance of the model is reached (e.g., AUC ≥ 0.90 on the held-out test set). Cases with discordant physician labels were excluded as a pre-specified quality-control step to reduce label noise during iterative training. A schematic of the active-learning sampling and blinded clinician adjudication workflow is provided in Supplementary Fig. 5.

Clinical adjudication (Operational Definition): PAS labels were assigned by clinicians via comprehensive, longitudinal chart reviews within the hospital information system (HIS), including laboratory and vital-sign trajectories, imaging, operative/laparoscopic records, medication administration, and microbiology/culture results. Specifically, after confirming that the patient (1) had a primary intra-abdominal infectious focus, (2) met pediatric SIRS criteria^30^, and (3) had concomitant acute gastrointestinal injury (AGI) of grade III-IV^31^, clinicians synthesized the above longitudinal evidence to determine PAS status. To prevent model-driven labeling, clinicians were blinded to model outputs during labeling. Notably, operative findings, imaging, and downstream treatment information were used only for clinical adjudication and transparency, and were not included as model inputs.

### Model construction and performance evaluation

In each labeling round, a random sample of 200 medical record IDs was selected from the dataset, and these IDs were then distributed to three physicians via the internal network encryption system (Fig. 1B). The labeled data were cleaned and then divided into a training set and a test set according to a random stratified sampling method at a ratio of 6:4.

The subsequent data were analyzed with logistic regression, random forest (RF), support vector machine (SVM), neural network (NN) and decision tree methodologies. These models were configured using the default parameters of each algorithm and underwent a 10-fold cross-validation process in the training set. The receiver operating characteristic (ROC) analysis and area under the ROC curve (AUC) were utilized to assess the predictive efficacy of the model and the algorithm that demonstrated the maximum average AUC was identified as optimal. Grid Search was then used to calibrate the hyper-parameters of the optimal model; the model that had been tuned was utilized as the diagnostic model for that particular round. Performance of the model was appraised in the test set and the labeling was terminated only when the AUC equal to or greater than 0.9. Otherwise, the subsequent round of labeling was initiated.

Following cessation of labeling, performance of the diagnostic model was evaluated using indicator importance ranking, AUC, and learning curve. In addition, SHapley Additive exPlanations (SHAP) was employed to assess the interpretability of the model^32^.

### Statistics

Model construction was performed using Python (version 3.9.12) and all statistical analyses were performed using R software (version 4.3.1). The *Streamlit* open-source Python library and Docker were used for building the ABSeD platform webserver. The Shapiro-Wilk test was used to determine the nature of data distribution. Statistical significance of baseline characteristics between the two groups was compared using the Student’s *t* test for normally distributed data; otherwise, the non-parametric Wilcoxon rank-sum test was used. Machine learning modeling and model evaluation were performed using the Python package “*sklearn*” (version 1.0.2). Heatmaps and forest plots were constructed using the “*pheatmap*” (version 1.0.12) and “*forestplot*” R packages (version 3.1.3). Confusion matrices were generated using the “*confusionMatrix*” function of the “*caret*” package of R software (version 6.0-94). Principal component analysis (PCA) was employed to reduce the dimensionality of the datasets and the analysis of similarities (ANOSIM) method utilized within the R package “*vegan*” (version 2.6-10) was used for comparison. The administrative boundaries of Zhejiang Province were retrieved from the “*gadm*” function of the “*geodata*” package in R (version 0.6-2).

### Ethics approval

This study was conducted in accordance with the Declaration of Helsinki and approved by the Institutional Review Board of the Children’s Hospital, Zhejiang University School of Medicine (retrospective: No. 2023-IRB-0221; prospective: No. 2025-IRB-0086). Yiwu Maternity and Children Hospital is a campus of the coordinating center and was covered by the same IRB. For this multicenter study, the coordinating-center IRB approval was formally acknowledged/accepted through research collaboration and data-sharing agreements by the local ethics committees of Shaoxing Maternal and Child Health Care Hospital, Jinhua Maternal and Child Health Care Hospital (JHFB-IEC-REG), Zhuji Maternity and Child Health Hospital, Wenling Maternal and Child Health Care Hospital, and Quzhou Maternal and Child Health Care Hospital. Informed consent was waived for the retrospective cohort because analyses used fully de-identified, routinely collected data. For the prospective validation cohort, the IRB approved a simplified consent procedure (brief information sheet and documented verbal parental/guardian consent), with child assent obtained when appropriate. The study is reported in accordance with the RECORD statement.

## Supplementary information

Supplementary information

## Data Availability

A specialized database for pediatric abdominal sepsis is currently being built. However, the author Zhigang Gao (ebwk@zju.edu.cn) can be contacted for de-identified participant data upon reasonable request. Applicants are required to complete some online training, such as the University of Miami Collaborative Institutional Training Program (CITI) “Data or Sample Only Research” course, and provide a Training completion report.

## Code availability

The datasets and materials used and/or analyzed during the current study are planted in https://github.com/CaoSuqi/ABSeD.

## References

[1] Rhee, C. et al. Incidence and trends of sepsis in us hospitals using clinical vs claims data, 2009-2014. *JAMA* **318**, 1241–1249, 10.1001/jama.2017.13836 (2017). 28903154 10.1001/jama.2017.13836 PMC5710396

[2] Singer, M. et al. The third international consensus definitions for sepsis and septic shock (sepsis-3). *JAMA* **315**, 801–810, 10.1001/jama.2016.0287 (2016). 26903338 10.1001/jama.2016.0287 PMC4968574

[3] Rudd, K. E. et al. Global, regional, and national sepsis incidence and mortality, 1990-2017: analysis for the Global Burden of Disease Study. *Lancet* **395**, 200–211, 10.1016/S0140-6736(19)32989-7 (2020). 31954465 10.1016/S0140-6736(19)32989-7 PMC6970225

[4] Black, R. E. et al. Global, regional, and national causes of child mortality in 2008: a systematic analysis. *Lancet* **375**, 1969–1987, 10.1016/S0140-6736(10)60549-1 (2010). 20466419 10.1016/S0140-6736(10)60549-1

[5] Roger, C. B., R A, B. F., Jerome, H., Abrams, G. R. B. J. & Cory Franklin, K. J. G. M. American College of Chest Physicians/Society of Critical Care Medicine Consensus Conference: definitions for sepsis and organ failure and guidelines for the use of innovative therapies in sepsis. *Crit. Care Med* **20**, 864–874 (1992). 1597042

[6] Levy, M. M. et al. 2001 SCCM/ESICM/ACCP/ATS/SIS international sepsis definitions conference. *Crit. Care Med* **31**, 1250–1256, 10.1097/01.CCM.0000050454.01978.3B (2003). 12682500 10.1097/01.CCM.0000050454.01978.3B

[7] Rhee, C., Gohil, S. & Klompas, M. Regulatory mandates for sepsis care-reasons for caution. *N. Engl. J. Med* **370**, 1673–1676, 10.1056/NEJMp1400276 (2014). 24738642 10.1056/NEJMp1400276 PMC4718398

[8] Watson, R. S. et al. The burden and contemporary epidemiology of sepsis in children. *Lancet Child Adolesc. Health* **8**, 670–681, 10.1016/S2352-4642(24)00140-8 (2024). 39142741 10.1016/S2352-4642(24)00140-8

[9] Sakr, Y. et al. Sepsis in intensive care unit patients: worldwide data from the intensive care over nations audit. *Open Forum Infect. Dis.* **5**, ofy313. 10.1093/ofid/ofy313 (2018). 30555852 10.1093/ofid/ofy313 PMC6289022

[10] Matics, T. J. & Sanchez-Pinto, L. N. Adaptation and validation of a pediatric sequential organ failure assessment score and evaluation of the sepsis-3 definitions in critically Ill children. *JAMA Pediatr.* **171**, e172352, 10.1001/jamapediatrics.2017.2352 (2017). 28783810 10.1001/jamapediatrics.2017.2352 PMC6583375

[11] Sanchez-Pinto, L. N. et al. Development and validation of the phoenix criteria for pediatric sepsis and septic shock. *JAMA* **331**, 675–686, 10.1001/jama.2024.0196 (2024). 38245897 10.1001/jama.2024.0196 PMC10900964

[12] Yang, Z., Cui, X. & Song, Z. Predicting sepsis onset in ICU using machine learning models: a systematic review and meta-analysis. *BMC Infect. Dis.* **23**, 635. 10.1186/s12879-023-08614-0 (2023). 37759175 10.1186/s12879-023-08614-0 PMC10523763

[13] Fleuren, L. M. et al. Machine learning for the prediction of sepsis: a systematic review and meta-analysis of diagnostic test accuracy. *Intensive Care Med* **46**, 383–400, 10.1007/s00134-019-05872-y (2020). 31965266 10.1007/s00134-019-05872-y PMC7067741

[14] Klassen, T. P., Hartling, L., Craig, J. C. & Offringa, M. Children are not just small adults: the urgent need for high-quality trial evidence in children. *PLoS Med* **5**, e172, 10.1371/journal.pmed.0050172 (2008). 18700813 10.1371/journal.pmed.0050172 PMC2504487

[15] Pierrakos, C., Velissaris, D., Bisdorff, M., Marshall, J. C. & Vincent, J. L. Biomarkers of sepsis: time for a reappraisal. *Crit. Care* **24**, 287. 10.1186/s13054-020-02993-5 (2020). 32503670 10.1186/s13054-020-02993-5 PMC7273821

[16] Zhang, W. Y. et al. Analysis and validation of diagnostic biomarkers and immune cell infiltration characteristics in pediatric sepsis by integrating bioinformatics and machine learning. *World J. Pediatr.* **19**, 1094–1103, 10.1007/s12519-023-00717-7 (2023). 37115484 10.1007/s12519-023-00717-7 PMC10533616

[17] Komorowski, M., Green, A., Tatham, K. C., Seymour, C. & Antcliffe, D. Sepsis biomarkers and diagnostic tools with a focus on machine learning. *EBioMedicine* **86**, 104394. 10.1016/j.ebiom.2022.104394 (2022). 36470834 10.1016/j.ebiom.2022.104394 PMC9783125

[18] Carrol, E. D. et al. Operationalizing appropriate sepsis definitions in children worldwide: considerations for the pediatric sepsis definition taskforce. *Pediatr. Crit. Care Med* **24**, e263–e271, 10.1097/PCC.0000000000003263 (2023). 37097029 10.1097/PCC.0000000000003263 PMC10226471

[19] Romaine, S. T. et al. Accuracy of a modified qSOFA score for predicting critical care admission in febrile children. *Pediatrics* **146**, 10.1542/peds.2020-0782 (2020). 10.1542/peds.2020-0782 PMC7786830 32978294

[20] Latremouille, S., Lam, J., Shalish, W. & Sant’Anna, G. Neonatal heart rate variability: a contemporary scoping review of analysis methods and clinical applications. *BMJ Open* **11**, e055209, 10.1136/bmjopen-2021-055209 (2021). 34933863 10.1136/bmjopen-2021-055209 PMC8710426

[21] Kemps, N. et al. The value of white blood cell count in predicting serious bacterial infections in children presenting to the emergency department: a multicentre observational study. *Arch. Dis. Child* **110**, 191–196, 10.1136/archdischild-2024-327493 (2025). 39332842 10.1136/archdischild-2024-327493 PMC11866293

[22] Rashwan, N. I., Hassan, M. H., Mohey El-Deen, Z. M. & Ahmed, A. E. Validity of biomarkers in screening for neonatal sepsis—a single center -hospital based study. *Pediatr. Neonatol.* **60**, 149–155, 10.1016/j.pedneo.2018.05.001 (2019). 29895470 10.1016/j.pedneo.2018.05.001

[23] Aygun, F., Kirkoc, R., Aygun, D. & Cam, H. Gamma glutamyl transferase and uric acid levels can be associated with the prognosis of patients in the pediatric intensive care unit. *Children (Basel)* **5**, 10.3390/children5110147 (2018). 10.3390/children5110147 PMC6262526 30380730

[24] Liu, Y., Chai, Y., Rong, Z. & Chen, Y. Prognostic value of ionized calcium levels in neonatal sepsis. *Ann. Nutr. Metab.* **76**, 193–200, 10.1159/000508685 (2020). 32756057 10.1159/000508685

[25] Chang, Z. et al. Clinical biomarker profiles reveals gender differences and mortality factors in sepsis. *Front Immunol.* **15**, 1413729. 10.3389/fimmu.2024.1413729 (2024). 38835774 10.3389/fimmu.2024.1413729 PMC11148215

[26] Barichello, T., Generoso, J. S., Singer, M. & Dal-Pizzol, F. Biomarkers for sepsis: more than just fever and leukocytosis-a narrative review. *Crit. Care* **26**, 14. 10.1186/s13054-021-03862-5 (2022). 34991675 10.1186/s13054-021-03862-5 PMC8740483

[27] Kainth, D., Prakash, S. & Sankar, M. J. Diagnostic performance of machine learning-based models in neonatal sepsis: a systematic review. *Pediatr. Infect. Dis. J.* **43**, 889–901, 10.1097/INF.0000000000004409 (2024). 39079037 10.1097/INF.0000000000004409

[28] Krack, A. T. et al. Leukopenia, neutropenia, and procalcitonin levels in young febrile infants with invasive bacterial infections. *Acad. Emerg. Med* **31**, 903–914, 10.1111/acem.14921 (2024). 38661246 10.1111/acem.14921

[29] Benzoni, N. S. et al. Temperature trajectory subphenotypes in oncology patients with neutropenia and suspected infection. *Am. J. Respir. Crit. Care Med* **207**, 1300–1309, 10.1164/rccm.202205-0920OC (2023). 36449534 10.1164/rccm.202205-0920OC PMC10595453

[30] Goldstein, B., Giroir, B., Randolph, A. & International Consensus Conference on Pediatric, S. International pediatric sepsis consensus conference: definitions for sepsis and organ dysfunction in pediatrics. *Pediatr. Crit. Care Med* **6**, 2–8, 10.1097/01.PCC.0000149131.72248.E6 (2005). 15636651 10.1097/01.PCC.0000149131.72248.E6

[31] Reintam Blaser, A. et al. Gastrointestinal function in intensive care patients: terminology, definitions and management. Recommendations of the ESICM Working Group on Abdominal Problems. *Intensive Care Med* **38**, 384–394, 10.1007/s00134-011-2459-y (2012). 22310869 10.1007/s00134-011-2459-y PMC3286505

[32] Lundberg, S. M. & Lee, S.-I. A unified approach to interpreting model predictions. *Nips'* **17**, 4768–4777 (2017).

