# Supplementary material for: Development and multicenter validation of an explainable machine learning diagnostic criteria for pediatric abdominal sepsis
Source: NPJ Digit Med. 2026 Mar 3;9:312. doi: 10.1038/s41746-026-02500-0 (PMC13077010; doi:10.1038/s41746-026-02500-0)
Supplement: Supplementary file 1 — Supplementary information [file 41746_2026_2500_MOESM1_ESM.pdf]

**Online supplement for Cao S, et al., Development and Multicenter Validation of an Explainable Machine Learning Diagnostic Criteria for Pediatric Abdominal Sepsis**

## Supplementary Tables

**Supplementary Table 1. Summary of basic nursing and laboratory test parameters in 743 children with abdominal diseases**

| Parameters | Control (N=430) | PAS (N=313) | *p* |
|---|---|---|---|
| Gender | | | |
| male | 271 (63%) | 175 (55.9%) | 0.060 |
| female | 159 (37%) | 138 (44.1%) | |
| Age (month) | 52.926 ± 51.258 | 88.265 ± 42.038 | <0.001 |
| Respiratory Rate (breaths/min) | 26.202 ± 5.090 | 24.805 ± 3.941 | <0.001 |
| Body Temperature (°C) | 36.945 ± 0.619 | 37.698 ± 0.751 | <0.001 |
| Body Weight (kg) | 18.204 ± 14.020 | 27.698 ± 14.792 | <0.001 |
| Heart Rate (beats/min) | 115.186 ± 16.886 | 118.073 ± 17.858 | 0.025 |
| $Cl^-$ (mmol/L) | 107.695 ± 3.893 | 104.691 ± 3.473 | <0.001 |
| Hematocrit HCT (%) | 37.811 ± 6.032 | 39.404 ± 4.826 | <0.001 |
| $K^+$ (mmol/L) | 3.988 ± 0.815 | 3.934 ± 1.227 | 0.522 |
| Lactate (mmol/L) | 1.914 ± 0.938 | 1.840 ± 0.915 | 0.321 |
| $Na^+$ (mmol/L) | 138.063 ± 3.898 | 135.600 ± 3.953 | <0.001 |
| Partial Pressure of Carbon Dioxide (mmHg) | 36.282 ± 6.037 | 34.518 ± 5.609 | <0.001 |
| PH | 7.396 ± 0.058 | 7.410 ± 0.043 | <0.001 |
| Partial Pressure of Oxygen (mmHg) | 94.629 ± 56.337 | 73.346 ± 38.152 | <0.001 |
| Standard Bicarbonate Concentration (mmol/L) | 22.457 ± 2.475 | 22.438 ± 2.410 | 0.921 |
| Standard Base Excess (mmol/L) | -2.409 ± 3.125 | -2.536 ± 3.160 | 0.614 |
| Oxygen Saturation (%) | 88.653 ± 14.958 | 86.200 ± 15.260 | 0.043 |
| Actual Base Excess (mmol/L) | -2.193 ± 3.098 | -2.153 ± 2.981 | 0.870 |
| $Ca^{2+}$ (mmol/L) | 1.159 ± 0.117 | 1.114 ± 0.087 | <0.001 |
| Absolute Neutrophil Count (x10^9/L) | 5.871 ± 5.120 | 12.799 ± 6.091 | <0.001 |
| Hemoglobin (g/L) | 119.555 ± 16.590 | 121.198 ± 13.452 | 0.137 |
| Absolute Lymphocyte Count (x10^9/L) | 4.358 ± 3.017 | 1.840 ± 1.130 | <0.001 |
| Mean Corpuscular Hemoglobin (pg) | 27.378 ± 2.495 | 27.694 ± 1.739 | 0.043 |
| Mean Corpuscular Hemoglobin Concentration (g/L) | 332.235 ± 12.563 | 333.920 ± 9.219 | 0.006 |
| Mean Corpuscular Volume (fL) | 82.336 ± 6.250 | 82.902 ± 4.403 | 0.148 |
| Platelet Count (x10^9/L) | 347.365 ± 127.605 | 324.275 ± 101.653 | 0.006 |
| Red Blood Cell Count (×10^12/L) | 4.389 ± 0.622 | 4.424 ± 0.503 | 0.427 |
| Red Cell Distribution Width (RDW) (%) | 13.513 ± 1.685 | 13.046 ± 1.089 | <0.001 |
| White Blood Cell Count (×10^9/L) | 11.187 ± 4.948 | 15.715 ± 6.328 | <0.001 |
| Absolute Basophil Count (×10^9/L) | 0.030 ± 0.024 | 0.027 ± 0.025 | 0.056 |
| Absolute Monocyte Count (×10^9/L) | 0.717 ± 0.445 | 0.963 ± 0.532 | <0.001 |
| Absolute Eosinophil Count (×10^9/L) | 0.212 ± 0.255 | 0.055 ± 0.098 | <0.001 |
| Albumin-to-Globulin Ratio | 1.791 ± 0.462 | 1.368 ± 0.368 | <0.001 |
| Albumin (g/L) | 41.861 ± 5.843 | 39.168 ± 5.626 | <0.001 |
| Alanine Aminotransferase (U/L) | 53.211 ± 111.444 | 17.282 ± 27.494 | <0.001 |

| Parameters | Control (N=430) | PAS (N=313) | *p* |
|---|---|---|---|
| Aspartate Aminotransferase (U/L) | 66.790 ± 99.779 | 34.997 ± 37.588 | <0.001 |
| Urea (mmol/L) | 4.021 ± 1.990 | 3.910 ± 1.675 | 0.458 |
| Calcium (mmol/L) | 2.397 ± 0.209 | 2.298 ± 0.138 | <0.001 |
| Cholesterol (mmol/L) | 4.234 ± 1.313 | 3.935 ± 1.055 | 0.001 |
| Creatine Kinase (U/L) | 132.575 ± 171.533 | 79.759 ± 95.219 | <0.001 |
| Creatine Kinase -MB (U/L) | 37.082 ± 34.826 | 28.921 ± 34.228 | 0.002 |
| Direct Bilirubin (µmol/L) | 6.026 ± 20.351 | 3.723 ± 10.609 | 0.056 |
| γ-Glutamyl Transferase (U/L) | 77.049 ± 208.852 | 30.901 ± 120.567 | <0.001 |
| Globulin (g/L) | 24.619 ± 5.858 | 29.447 ± 4.710 | <0.001 |
| Indirect Bilirubin (µmol/L) | 13.937 ± 33.862 | 9.850 ± 11.323 | 0.026 |
| Magnesium (mmol/L) | 0.875 ± 0.120 | 0.859 ± 0.110 | 0.069 |
| Phosphorus (mmol/L) | 1.730 ± 0.410 | 1.532 ± 0.494 | <0.001 |
| Total Bile Acids (µmol/L) | 12.756 ± 30.901 | 6.626 ± 18.490 | 0.001 |
| Total Bilirubin (µmol/L) | 19.962 ± 46.764 | 13.572 ± 21.123 | 0.017 |
| Triglycerides (mmol/L) | 1.078 ± 0.627 | 0.930 ± 0.450 | <0.001 |
| Total Protein (g/L) | 66.525 ± 8.873 | 68.675 ± 7.809 | <0.001 |
| Uric Acid (µmol/L) | 277.215 ± 114.288 | 295.418 ± 108.641 | 0.036 |
| Lipase (U/L) | 50.557 ± 186.133 | 36.630 ± 209.511 | 0.368 |
| Amylase (U/L) | 53.234 ± 88.229 | 46.320 ± 30.747 | 0.225 |
| High-Sensitivity C-Reactive Protein (mg/L) | 15.022 ± 29.141 | 84.844 ± 53.739 | <0.001 |
| Prealbumin (mg/L) | 160.201 ± 54.290 | 118.130 ± 47.460 | <0.001 |

**Supplementary Table 2. Clinical characterization of adjudicated PAS vs. controls.**

| Parameters | Control (N=430) | PAS (N=313) | *p* |
|---|---|---|---|
| Surgery | | | |
| Yes | 119 (27.7%) | 117 (37.4%) | 0.060 |
| No | 311 (72.3%) | 196 (62.6%) | |
| Prolonged escalation antibiotic therapy (>7 days) | | | |
| Yes | 108 (25.1%) | 104 (33.2%) | 0.020 |
| No | 322 (74.9%) | 209 (66.8%) | |
| Intra-abdominal abscess | | | |
| Yes | 52 (12.1%) | 112 (35.8%) | <0.001 |
| No | 378 (87.9%) | 201 (64.2%) | |
| Hospital stay time (days) | 9.143 [4.090, 16.132] | 11.000 [8.942, 15.770] | <0.001 |

Notes*: The variables in this table are reported to increase transparency of the clinician-adjudicated PAS construct and to provide objective clinical anchors (treatment intensity and disease severity proxies). These variables were not used as predictors in ABSeD.

**Supplementary Table 3. Summary of the univariate logistic regression analysis.**

| Parameters | Odds Ratio (OR) | lower | upper | *p* |
|---|---|---|---|---|
| Gender | 1.344 | 0.999 | 1.809 | 0.051 |
| Age (month) | 1.015 | 1.012 | 1.018 | <0.001 |
| Respiratory Rate (breaths/min) | 0.932 | 0.899 | 0.965 | <0.001 |
| Body Temperature (°C) | 4.65 | 3.618 | 6.067 | <0.001 |
| Body Weight (kg) | 1.047 | 1.035 | 1.06 | <0.001 |
| Heart Rate (beats/min) | 1.01 | 1.001 | 1.018 | 0.026 |
| $Cl^-$ (mmol/L) | 0.798 | 0.757 | 0.838 | <0.001 |
| Hematocrit HCT (%) | 1.054 | 1.024 | 1.087 | <0.001 |
| $K^+$ (mmol/L) | 0.946 | 0.783 | 1.108 | 0.511 |
| Lactate (mmol/L) | 0.916 | 0.769 | 1.087 | 0.321 |
| $Na^+$ (mmol/L) | 0.848 | 0.81 | 0.887 | <0.001 |
| Partial Pressure of Carbon Dioxide (mmHg) | 0.949 | 0.923 | 0.976 | <0.001 |
| PH | 193.19 | 8.587 | 4847.214 | 0.001 |
| Partial Pressure of Oxygen (mmHg) | 0.991 | 0.987 | 0.994 | <0.001 |
| Standard Bicarbonate Concentration (mmol/L) | 0.997 | 0.935 | 1.063 | 0.921 |
| Standard Base Excess (mmol/L) | 0.987 | 0.939 | 1.038 | 0.614 |
| Oxygen Saturation (%) | 0.989 | 0.979 | 1 | 0.044 |
| Actual Base Excess (mmol/L) | 1.004 | 0.954 | 1.058 | 0.870 |
| $Ca^{2+}$ (mmol/L) | 0.015 | 0.003 | 0.071 | <0.001 |
| Absolute Neutrophil Count (x10^9/L) | 1.231 | 1.193 | 1.272 | <0.001 |
| Hemoglobin (g/L) | 1.007 | 0.998 | 1.017 | 0.151 |
| Absolute Lymphocyte Count (x10^9/L) | 0.517 | 0.454 | 0.582 | <0.001 |
| Mean Corpuscular Hemoglobin (pg) | 1.069 | 0.999 | 1.145 | 0.057 |
| Mean Corpuscular Hemoglobin Concentration (g/L) | 1.014 | 1 | 1.028 | 0.046 |
| Mean Corpuscular Volume (fL) | 1.019 | 0.992 | 1.047 | 0.171 |
| Platelet Count (x10^9/L) | 0.998 | 0.997 | 1 | 0.009 |
| Red Blood Cell Count (×10^12/L) | 1.112 | 0.847 | 1.467 | 0.446 |
| Red Cell Distribution Width (%) | 0.765 | 0.668 | 0.865 | <0.001 |
| White Blood Cell Count (×10^9/L) | 1.155 | 1.121 | 1.191 | <0.001 |
| Absolute Basophil Count (×10^9/L) | 0.002 | 0 | 1.112 | 0.057 |
| Absolute Monocyte Count (×10^9/L) | 3.12 | 2.209 | 4.483 | <0.001 |
| Absolute Eosinophil Count (×10^9/L) | 0.001 | 0 | 0.004 | <0.001 |
| Albumin-to-Globulin Ratio | 0.052 | 0.029 | 0.088 | <0.001 |
| Albumin (g/L) | 0.922 | 0.896 | 0.948 | <0.001 |
| Alanine Aminotransferase (U/L) | 0.983 | 0.974 | 0.99 | <0.001 |
| Aspartate Aminotransferase (U/L) | 0.988 | 0.982 | 0.992 | <0.001 |
| Urea (mmol/L) | 0.968 | 0.883 | 1.056 | 0.467 |
| Calcium (mmol/L) | 0.034 | 0.012 | 0.092 | <0.001 |
| Cholesterol (mmol/L) | 0.796 | 0.687 | 0.915 | 0.002 |

| Parameters | Odds Ratio (OR) | lower | upper | *p* |
|---|---|---|---|---|
| Creatine Kinase (U/L) | 0.993 | 0.991 | 0.996 | <0.001 |
| Creatine Kinase -MB (U/L) | 0.992 | 0.987 | 0.997 | 0.003 |
| Direct Bilirubin (μmol/L) | 0.988 | 0.973 | 1 | 0.098 |
| γ-Glutamyl Transferase (U/L) | 0.997 | 0.995 | 0.999 | 0.002 |
| Globulin (g/L) | 1.183 | 1.145 | 1.225 | <0.001 |
| Indirect Bilirubin (μmol/L) | 0.992 | 0.982 | 0.999 | 0.066 |
| Magnesium (mmol/L) | 0.292 | 0.075 | 1.094 | 0.071 |
| Phosphorus (mmol/L) | 0.35 | 0.237 | 0.506 | <0.001 |
| Total Bile Acids (μmol/L) | 0.987 | 0.975 | 0.995 | 0.009 |
| Total Bilirubin (μmol/L) | 0.994 | 0.988 | 0.999 | 0.042 |
| Triglycerides (mmol/L) | 0.6 | 0.441 | 0.804 | 0.001 |
| Total Protein (g/L) | 1.031 | 1.012 | 1.051 | 0.001 |
| Uric Acid (μmol/L) | 1.001 | 1 | 1.003 | 0.038 |
| Lipase (U/L) | 1 | 0.998 | 1 | 0.377 |
| High-Sensitivity C-Reactive Protein (mg/L) | 1.039 | 1.034 | 1.045 | <0.001 |
| Prealbumin (mg/L) | 0.984 | 0.981 | 0.987 | <0.001 |

**Supplementary Table 4. Data collection and model prediction performance in different hospitals**

| | Case number | | Accuracy | Precision | F1 | Recall |
|---|---|---|---|---|---|---|
| | PAS | Control | | | | |
| All cases | 80 | 228 | 0.873 | 0.924 | 0.914 | 0.904 |
| CHZJU | 16 | 36 | 0.827 | 0.846 | 0.88 | 0.917 |
| JHMC | 26 | 33 | 0.831 | 0.848 | 0.848 | 0.848 |
| QZMC | 2 | 0 | Two PAS cases were correctly identified | | | |
| SXMC | 2 | 83 | 0.953 | 0.988 | 0.976 | 0.964 |
| YWMC | 30 | 70 | 0.85 | 0.923 | 0.889 | 0.857 |
| ZJMC | 4 | 2 | 0.833 | 1 | 0.667 | 0.5 |
| WLMC | 0 | 4 | Four control cases were correctly identified | | | |

CHZJU: Children's Hospital Zhejiang University School of Medicine; JHMC: Jinhua Maternal and Child Health Care Hospital; QZMC: Quzhou Maternal and Child Health Care Hospital; SXMC: Shaoxing Maternal and Child Health Care Hospital; YWMC: Yiwu Maternal and Child Health Care Hospital; ZJMC: Zhuji Maternal and Child Health Care Hospital; WLMC: Wenling Maternal and Child Health Care Hospital

## Supplementary Figures

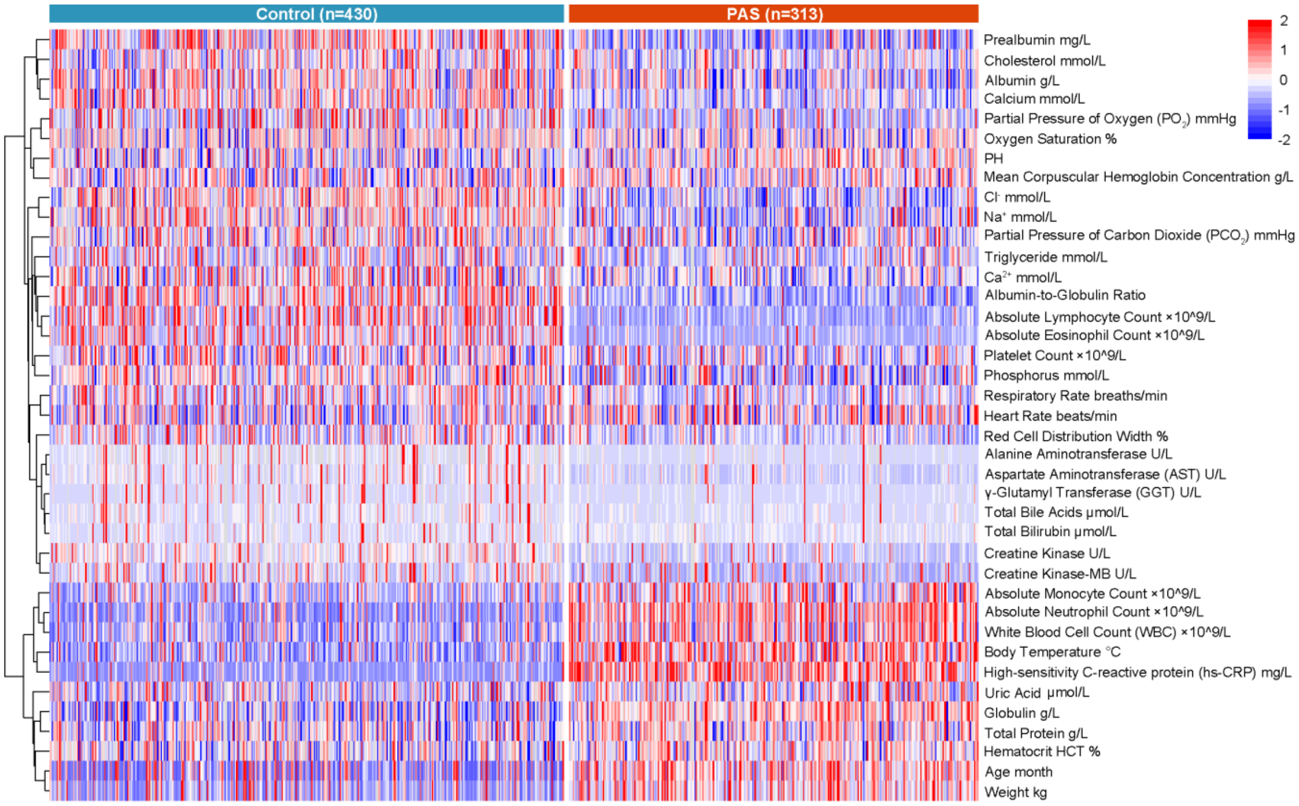


**Supplementary Figure 1. Heatmap exhibits the normalized characteristics of children with abdominal diseases. The x-axis represents 743 children, while the y-axis represents 39 potential PAS diagnostic markers that were screened by univariate logistic regression.**

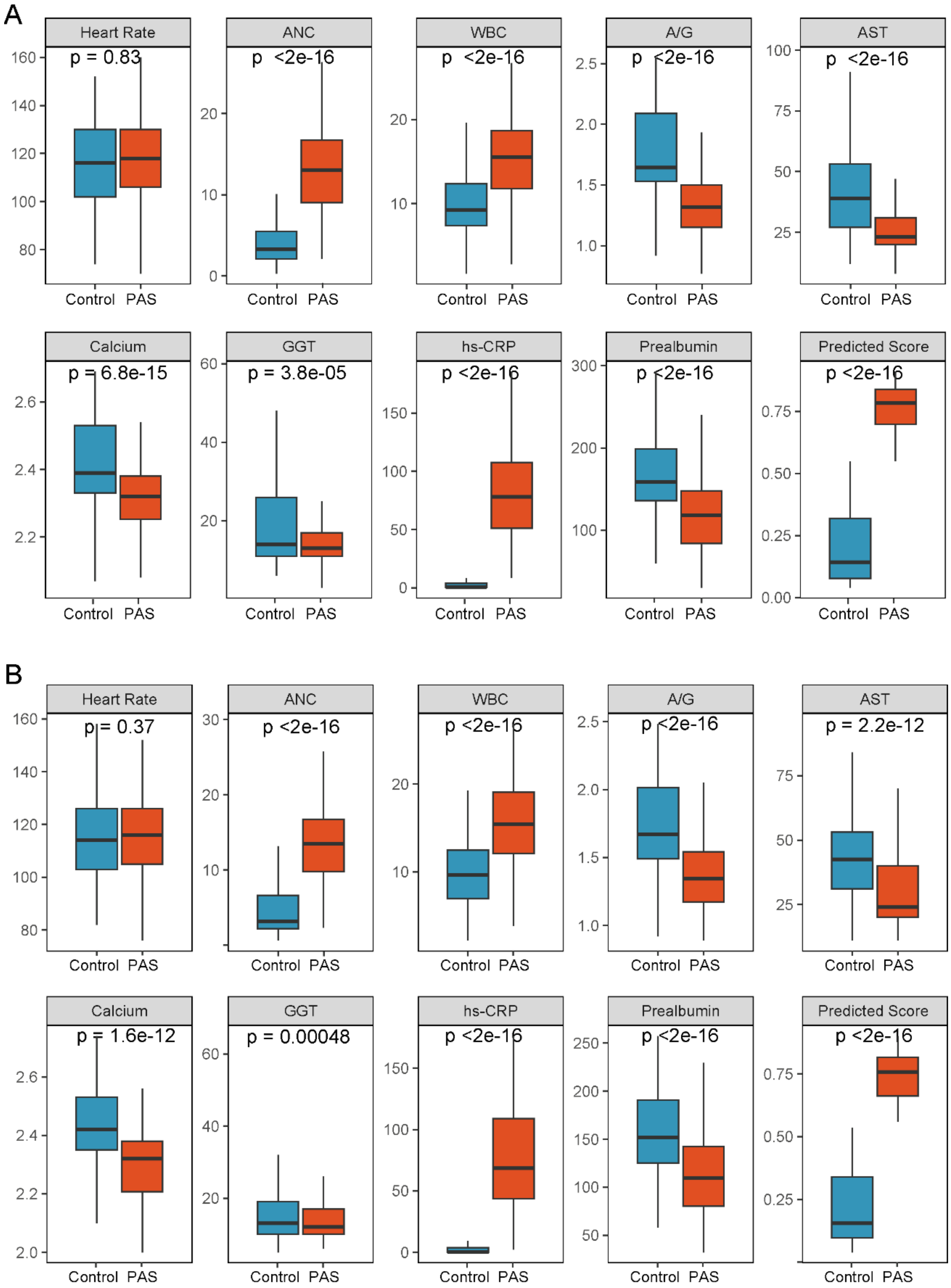


**Supplementary Figure 2. Boxplots presenting the differences in 9 markers and predicted score between PAS and control groups in (A) training set and (B) test set.**

ANC: absolute neutrophil count; WBC: white blood cell count; A/G: albumin-to-globulin ratio; AST: aspartate aminotransferase; GGT: γ-glutamyl transferase; hs-CRP: high-sensitivity C-reactive protein.

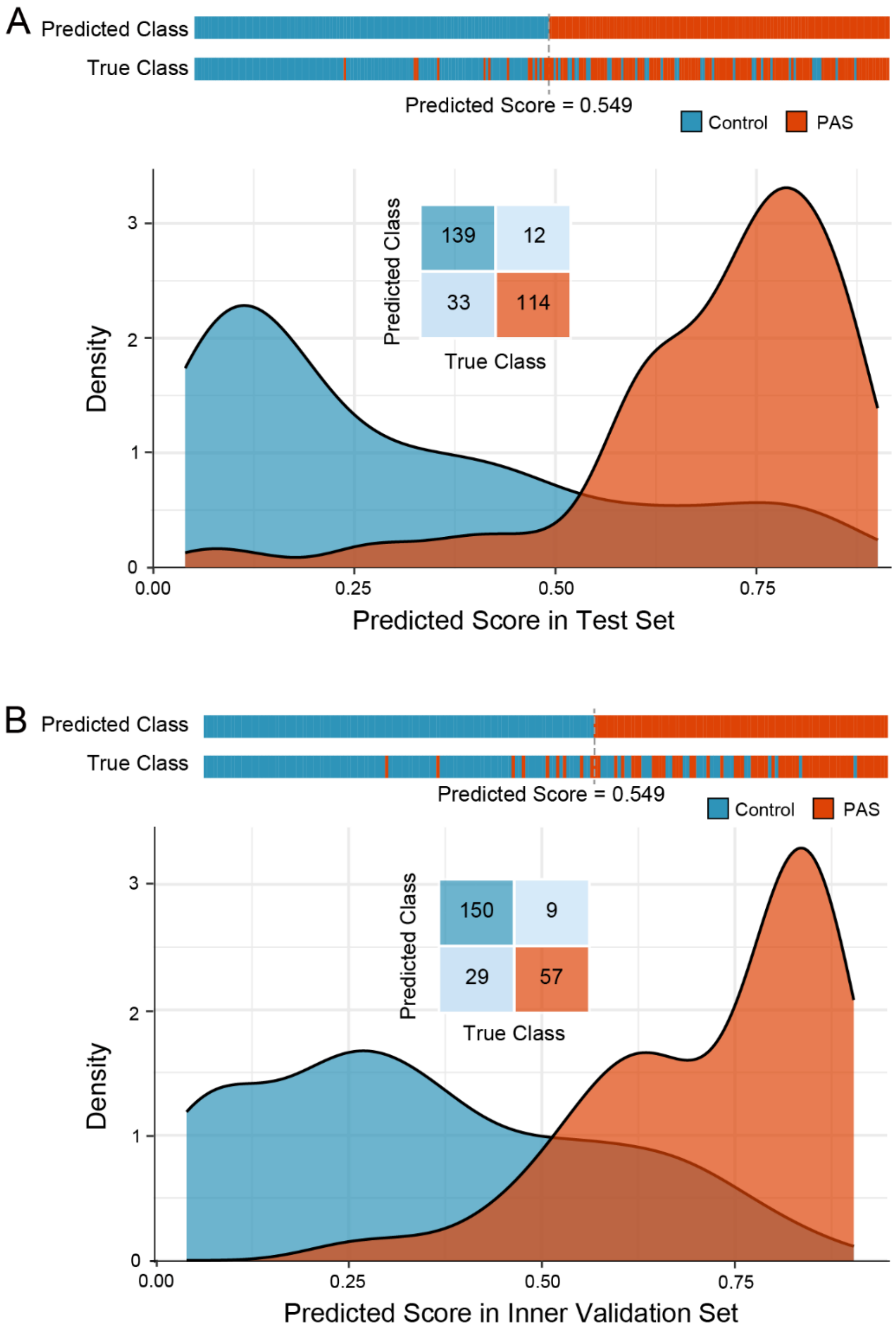


**Supplementary Figure 3. Distribution of predicted scores and confusion matrix of ABSeD model in the PAS and control groups in the (A) test set and (B) inner validation set.**

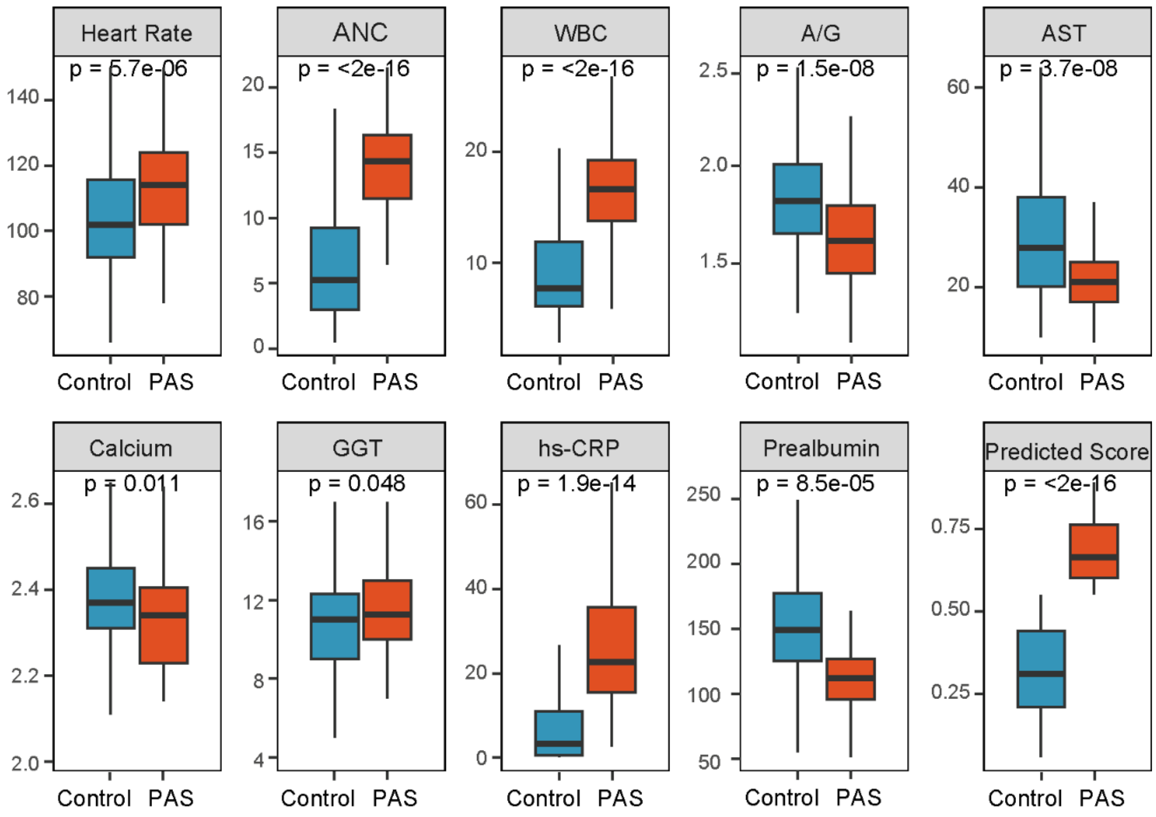


**Supplementary Figure 4. Boxplots present the differences of 9 markers and predicted score between PAS and control groups in the external validation set.**

ANC: absolute neutrophil count; WBC: white blood cell count; A/G: albumin-to-globulin ratio; AST: aspartate aminotransferase; GGT: γ-glutamyl transferase; hs-CRP: high-sensitivity C-reactive protein.

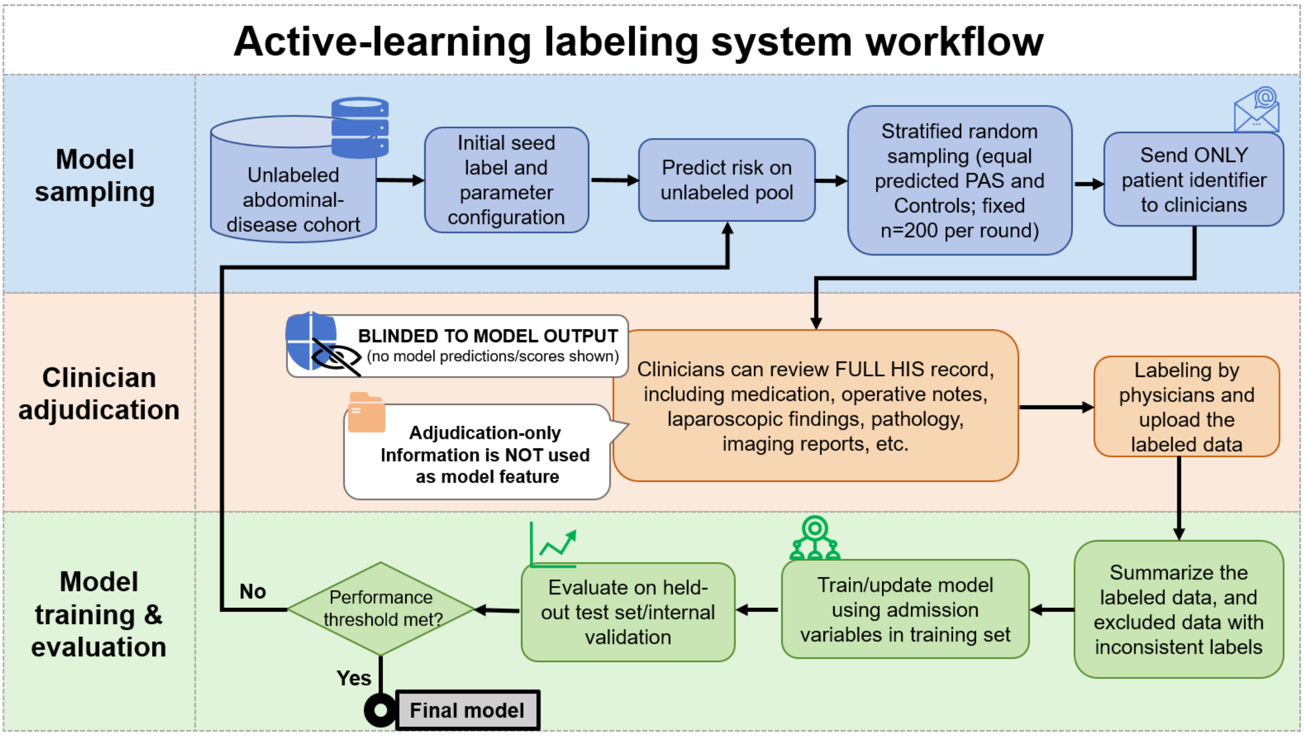


**Supplementary Figure 5. Active-learning sampling and clinician adjudication workflow.**
